# Structure Peculiarities of Micro- and Nanocrystalline Perovskite Ferrites La_1−*x*_Sm_*x*_FeO_3_

**DOI:** 10.1186/s11671-017-1946-7

**Published:** 2017-02-27

**Authors:** O. B. Pavlovska, L. O. Vasylechko, I. V. Lutsyuk, N. M. Koval, Ya A. Zhydachevskii, A. Pieniążek

**Affiliations:** 1 Semiconductor Electronics Department of Lviv Polytechnic National University, 12 Bandera Street, 79013 Lviv, Ukraine; 2 Department of Chemical Technology of Silicates of Lviv Polytechnic National University, 12 Bandera Street, 79013 Lviv, Ukraine; 3 Department of Ecological Safety and Nature Protection Activity of Lviv Polytechnic National University, 12 Bandera Street, 79013 Lviv, Ukraine; 4 Institute of Physics, Polish Academy of Sciences, Al. Lotników 32/46, 02-668 Warsaw, Poland

**Keywords:** Mixed rare earth ferrites, Perovskites, Crystal structure, Solid solution, Lattice crossover

## Abstract

Micro- and nanocrystalline lanthanum-samarium ferrites La_1−*x*_Sm_*x*_FeO_3_ with orthorhombic perovskite structure were obtained by using both solid state reactions (*x* = 0.2, 0.4, 0.6 and 0.8) and sol-gel synthesis (*x* = 0.5) techniques. Obtained structural parameters of both series of La_1−*x*_Sm_*x*_FeO_3_ are in excellent agreement with the “pure” LaFeO_3_ and SmFeO_3_ compounds, thus proving formation of continuous solid solution in the LaFeO_3_–SmFeO_3_ system. Peculiarity of La_1−*x*_Sm_*x*_FeO_3_ solid solution is divergence behaviour of unit cell dimensions with increasing *x*: systematic decrease of the *a* and *c* lattice parameters is accompanied with increasing *b* parameter. Such behaviour of the unit cell dimensions in La_1−*x*_Sm_*x*_FeO_3_ series led to crossover of the *a* and *c* perovskite lattice parameters and formation of dimensionally tetragonal structure near *x* = 0.04. Linear decrease of the unit cell volume of La_1−*x*_Sm_*x*_FeO_3_ with decreasing *x* according with the Vegard’s rule indicate absence of short-range ordering of *R*-cations in the LaFeO_3_–SmFeO_3_ system.

## Background

The interest in the rare earth ferrites *R*FeO_3_ (*R* = rare earths) is stimulated by their unique properties, such as high electrical conductivity, specific magnetic properties including spin reorientation phenomena, as well as significant electrochemical and catalytic activity. *R*FeO_3_-based materials are used as electrode materials in solid oxide fuel cells [1, 2], as membranes for gases separation, sensory materials and catalysts [3–6], and as magnetic and multiferroic materials [7–10].

Among *R*FeO_3_ compounds, lanthanum and samarium orthoferrites are two of the most studied materials because of combination of several intrigue properties [10–13]. At the ambient conditions, both LaFeO_3_ and SmFeO_3_ display the orthorhombic perovskite structure isotypic with GdFeO_3_ [14, 15]. In situ high-resolution X-ray synchrotron and neutron powder diffraction examination revealed no structural changes in SmFeO_3_ in the temperature range of 300–1173 K [16], whereas LaFeO_3_ undergoes the first-order orthorhombic-to-rhombohedral structural phase transition at 1253–1260 K [17–19]. Lattice expansion of LaFeO_3_ and SmFeO_3_ shows non-linear and strongly anisotropic thermal behaviour: in both compounds relative expansion in *b*-direction is much lower than in *a*- and *c*-directions [16, 18–20]. As a result, lattice parameter crossovers occur in LaFeO_3_ at 750–950 K [18–20]. Subtle anomalies in the lattice expansion detected in LaFeO_3_ and SmFeO_3_ are associated with antiferromagnetic—to paramagnetic phase transition occurred in these compounds at 735 and 670 K, respectively [11, 12, 16, 21]. In LaFeO_3_, such anomalies are reflected in non-linear lattice expansion across the magnetic phase transition at the Néel temperature 735 K [18] and in the step of dilatometric thermal expansion coefficient at 723 ± 50 K. In SmFeO_3_, the *b* parameter exhibits a small anomalous kink around 670 K that is indicative for magnetoelastic coupling at the magnetic ordering temperature *T*_*N*_ [16]. Similar sign of magnetoelastic coupling was recently detected in the mixed ferrite system SmFeO_3_–PrFeO_3_, in which subtle maxima at the thermal expansion curves were observed in Sm_0.5_Pr_0.5_FeO_3_ at around 670 K [22].

The aim of the present work is synthesis of phase pure micro- and nanocrystalline powders of lanthanum-samarium orthoferrites La_1−*x*_Sm_*x*_FeO_3_ and their detailed structural investigation in whole concentration range.

## Methods

Micro- and nanocrystalline samples of the mixed lanthanum-samarium ferrites were prepared by two different experimental routes. Samples with nominal compositions La_1−*x*_Sm_*x*_FeO_3_ (*x* = 0.2, 0.4, 0.6 and 0.8) were obtained by solid state reactions technique. Precursor oxides La_2_O_3_, Sm_2_O_3_ and Fe_2_O_3_ were ball-milled in ethanol for 5 h, dried, pressed into pellets and annealed in air at 1473 K for 40 h with one intermediate regrinding. The synthesis of La_1−*x*_Sm_*x*_FeO_3_ can be presented by following reaction scheme:

$$ \left(1- x\right){\mathrm{La}}_2{\mathrm{O}}_3+ x{\mathrm{Sm}}_2{\mathrm{O}}_3+{\mathrm{Fe}}_2{\mathrm{O}}_3\to 2{\mathrm{La}}_{1- x}{\mathrm{Sm}}_x{\mathrm{Fe}\mathrm{O}}_3 $$

For a preparation of nanocrystalline powders of nominal composition, La_0.5_Sm_0.5_FeO_3_ sol-gel citrate method was used. Crystalline salts La(NO_3_)_3_ · 6H_2_O (99.99%, Alfa Aesar), Sm(NO_3_)_3_ · 6H_2_O (ACS, Alfa Aesar) and Fe(NO_3_)_3_ · 9H_2_O (ACS, Alfa Aesar) and citric acid (CC) were dissolved in water and mixed in the molar ratio of *n*(La^3+^):*n*(Sm^2+^):*n*(Fe^3+^):*n*(CC) = 1:0.5:0.5:4 according to the nominal composition of the sample. Prepared solution was gelled at ~90 °C and heat treated at 1073 K for 2 h. After X-ray diffraction (XRD) examination, the part of the powder was additionally annealed at 1173 K for 2 h and then at 1473 K for 4 h. Thus three La_0.5_Sm_0.5_FeO_3_ specimens, synthesized at different conditions, were obtained.

X-ray phase and structural characterization of the samples were performed by using Huber imaging plate Guinier camera G670 (Cu *K*_α1_ radiation, *λ* = 1.54056 Å). Spot-check examination of the cationic composition was performed by energy dispersive X-ray fluorescence (EDXRF) analysis by using XRF Analyzer Expert 3L. Based on the experimental powder diffraction data, the unit cell dimensions and positional and displacement parameters of atoms in the La_1−*x*_Sm_*x*_FeO_3_ structures were derived by full profile Rietveld refinement technique using software package WinCSD [23]. This programme package was also used for the evaluation of microstructural parameters of La_0.5_Sm_0.5_FeO_3_ powders from angular dependence of the Bragg’s maxima broadening. Average grain size, *D*_ave_ and microstrains <ε> = <Δ*d*>/*d* were derived both by full profile Rietveld refinement and by using Williamson-Hall analysis, which allows to separate the effect of size and strain broadening due to their different dependence on the scattering angle. In both cases, LaB_6_ external standard was used for the correction of instrumental broadening. The morphology of sol-gel derived La_0.5_Sm_0.5_FeO_3_ samples synthesized at different conditions was investigated by means of Hitachi SU-70 scanning electron microscope.

## Results and Discussion

**Fig. 1:**
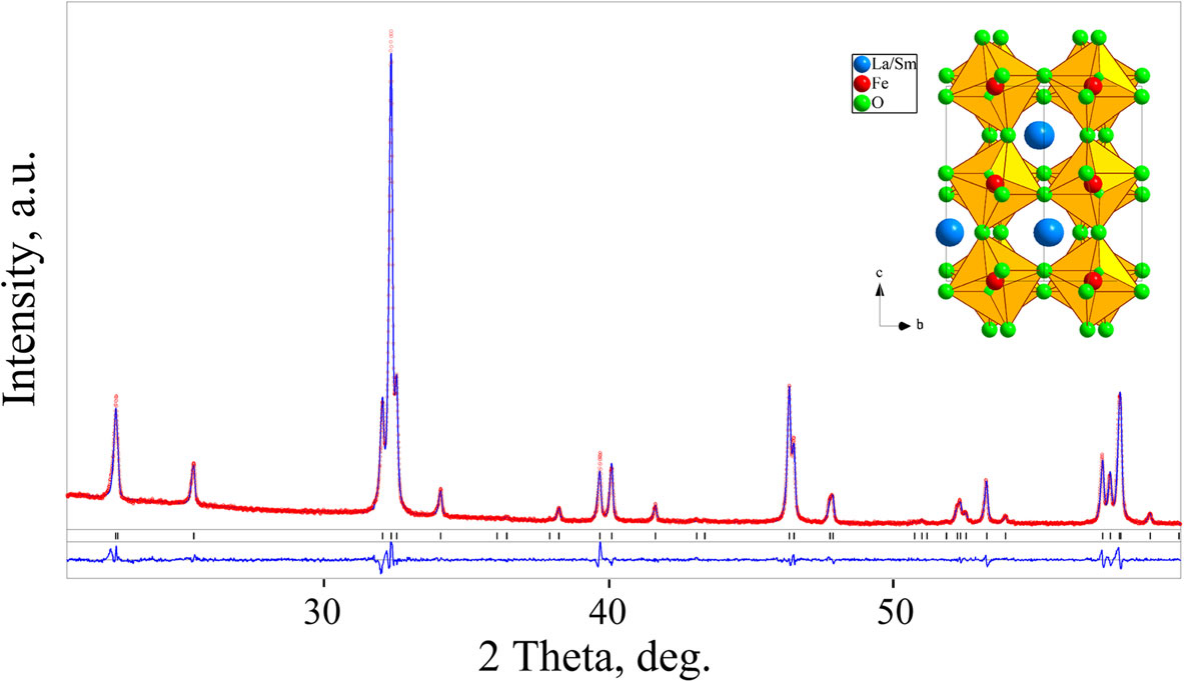
Graphical results of Rietveld refinement of the La_0.6_Sm_0.4_FeO_3_ structure. Experimental X-ray powder diffraction pattern (*red dots*) is shown in comparison with the calculated pattern (*blue line*). The difference between measured and calculated profiles is shown as a curve below the diagrams. *Short vertical bars* indicate the positions of diffraction maxima in space group *Pbnm. Inset* shows the view of the structure as corner-shared FeO_6_ octahedra with La/Sm species located between them

X-ray phase and structural analysis revealed that all La_1−*x*_Sm_*x*_FeO_3_ samples synthesized by solid state method at 1473 K for 40 h adopt orthorhombic perovskite structure isotypic with GdFeO_3_. No additional crystalline phases were found. Full profile Rietveld refinement, performed in space group *Pbnm*, shows excellent agreement between experimental and calculated diffraction patterns (Fig. 1) thus proving phase purity and crystal structure of the samples.

**Fig. 2:**
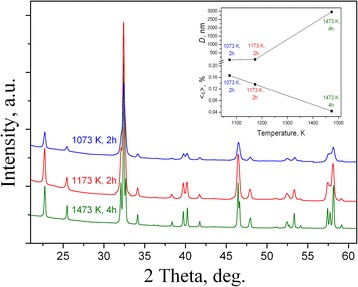
XRD patterns of La_0.5_Sm_0.5_FeO_3_ samples obtained at different conditions. *Inset* shows evolution of microstructural parameters vs the temperature and duration of heat treatment of the specimens

X-ray powder diffraction examination of sol-gel derived La_0.5_Sm_0.5_FeO_3_ sample shows that even short-term heat treatment of the dried xerogel at 1073 K for 2 h led to formation of pure perovskite structure, without any traces of precursor components or other parasitic phases (Fig. 2). Substantial broadening of the diffraction maxima observed at the XRD pattern of La_0.5_Sm_0.5_FeO_3_@1073 sample clearly indicates the nanoscale particle size of the as-obtained product.

**Fig. 3:**
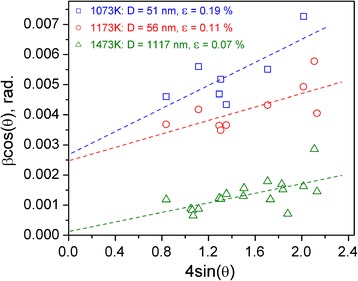
Graphical results of the Williamson-Hall analysis of La_0.5_Sm_0.5_FeO_3_ microstructure parameters

Indeed, evaluation of microstructural parameters of the La_0.5_Sm_0.5_FeO_3_@1073 sample from the analysis of the XRD profile broadening by full profile Rietveld technique lead to the average grain size *D*_ave_ = 78 nm and microstrains <ε> = < Δ*d*>/*d* = 0.17%. Additional heat treatment of the sample at 1173 and 1473 K does not affects on the phase composition and crystal structure parameters of the sample; the main changes occurs in the microstructural parameters, as it is evidenced from the significant narrowing of the Bragg’s maxima, especially pronounced in La_0.5_Sm_0.5_FeO_3_@1473 sample (Fig. 2). Evolution of the average grain sizes and microstrains in La_0.5_Sm_0.5_FeO_3_ specimens vs synthesis temperature (Fig. 2, inset) clearly shows systematic increase of the average grain sizes, *D*_ave_, accompanied with simultaneous reducing of the lattice strains. The *D*_ave_ values increases weakly from 78 to 103 nm after additional annealing at 1173 K for 2 h, whereas further heat treatment of the sample at 1473 K for 4 h lead to the drastic increase of the crystallite size up to >2000 nm. Similar evolution of the microstructural parameters of La_0.5_Sm_0.5_FeO_3_ was obtained from the Williamson-Hall analysis (Fig 3). No obvious selective *hkl-*dependent peak broadening was observed for the samples heat treated at different temperatures.

**Fig. 4:**
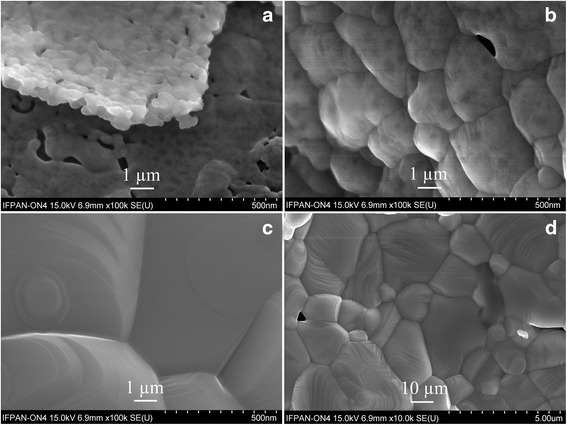
SEM pictures of La_0.5_Sm_0.5_FeO_3_ synthesized at 1073 K (**a**), 1173 K (**b**) and 1473 K (**c**, **d**)

Scanning electronic microscopy of the pristine La_0.5_Sm_0.5_FeO_3_, obtained at 1073 K, revealed sheets-like morphology of the powder consisting of the particles of irregular form with linear dimensions 200–500 nm (Fig. 4a). Taking into account that average grain size of La_0.5_Sm_0.5_FeO_3_@1073 sample derived from XRD data is 51–73 nm, it is evident that the particles observed at SEM picture of the sample consist of several smaller crystallites. SEM examination also confirms the temperature evolution of microstructural parameters of La_0.5_Sm_0.5_FeO_3_, derived from the X-ray powder diffraction data. As it is evidenced from Fig. 4b, additional heat treatment of the La_0.5_Sm_0.5_FeO_3_ sample at 1173 K for 2 h leads to the particle agglomeration and formation of 1–5 μm agglomerates, consisting of several submicron particles. Finally, further heat treatment of the sample at 1473 K for 4 h lead to coalescence of small grains and particles and formation of 10–50 μm crystallites with clear signs of the facet growth (Fig. 4c, d).

Refined values of unit cell dimensions and positional and displacement parameters of atoms for sol-gel-derived La_0.5_Sm_0.5_FeO_3_ samples, heat treated at 1073 and 1473 K, as well as the La_1−*x*_Sm_*x*_FeO_3_ specimens with *x* = 0.2, 0.4, 0.6 and 0.8, obtained by traditional ceramic technology are presented in Table 1.

**Table 1:** Lattice parameters, coordinates and displacement parameters of atoms in La_1−*x*_Sm_*x*_FeO_3_ structures

Atoms, sites	Parameters, residuals	*x* = 0.2	*x* = 0.4	*x* = 0.5^a^, 1073 K	*x* = 0.5^a^, 1473 K	*x* = 0.6	*x* = 0.8
	*a*, Å	5.5284(8)	5.4921(5)	5.477(1)	5.4750(3)	5.4618(3)	5.4319(9)
	*b*, Å	5.5694(8)	5.5759(5)	5.565(1)	5.5731(3)	5.5839(3)	5.5914(9)
	*c*, Å	7.833(1)	7.8000(7)	7.782(2)	7.7825(4)	7.7717(4)	7.742(2)
	*V,* Å^3^	241.19(8)	238.86(7)	237.2(2)	237.46(4)	237.02(4)	235.1(2)
La/Sm, 4*c*	*x*	−0.0087(3)	−0.0096(3)	−0.0032(10)	−0.0095(3)	−0.0105(2)	−0.0105(4)
	*y*	0.0354(2)	0.0432(1)	0.0421(2)	0.0448(1)	0.0481(1)	0.0523(2)
	*z*	1/4	1/4	1/4	1/4	1/4	1/4
	*B*_iso_, Å^2^	0.53(2)	0.51(2)	0.68(3)	0.54(2)	0.71(2)	0.71(3)
Fe, 4*b*	*x*	0	0	0	0	0	0
	*y*	1/2	1/2	1/2	1/2	1/2	1/2
	*z*	0	0	0	0	0	0
	*B*_iso_, Å^2^	1.15(4)	1.27(4)	1.38(6)	0.81(4)	0.99(3)	0.89(6)
O1, 4*c*	*x*	0.051(3)	0.080(2)	0.080(3)	0.0820(15)	0.0943(12)	0.087(2)
	*y*	0.5028(13)	0.4703(14)	0.466(2)	0.4764(13	0.4632(11)	0.468(2)
	*z*	1/4	1/4	1/4	1/4	1/4	1/4
	*B*_iso_, Å^2^	0.9(4)	2.4(3)	0.6(2)	2.4(2)	1.8(2)	0.5(3)
O2, 8*d*	*x*	−0.314(2)	−0.3047(12)	−0.315(2)	−0.2908(12)	−0.2837(9)	−0.3078(15)
	*y*	0.284(2)	0.2753(13)	0.274(2)	0.2865(12)	0.2863(9)	0.284(2)
	*z*	0.0392(15)	0.0475(9)	0.054(2)	0.0467(8)	0.0543(6)	0.0519(11)
	*B*_iso_, Å^2^	2.1(3)	1.5(2)	0.6(2)	1.5(2)	0.89(13)	0.6(3)
	*R*_*I*_	0.071	0.056	0.104	0.046	0.053	0.089
	*R*_*P*_	0.144	0.116	0.183	0.124	0.106	0.181

^a^Synthesized by sol-gel method

**Fig. 5:**
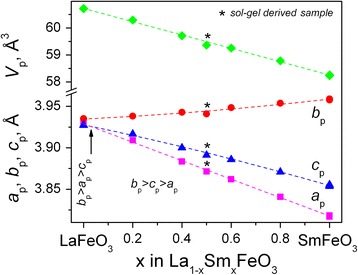
Concentration dependencies of unit cell dimensions of La_1−*x*_Sm_*x*_FeO_3_. Orthorhombic lattice parameters and unit cell volume are normalized to the perovskite ones as follows: *a*_*p*_ *= a*_*o*_ */√2, b*_*p*_ *= b*_*o*_ */√2, c*_*p*_ *= c*_*o*_/2, *V*_*p*_ *= V*_*o*_/4*.* The *dashed lines* are polynomial fits: *a*_*p*_ *=* 3.9295(9) − 0.115(4) × *x* + 0.004(4) × *x* ^*2*^ *; b*_*p*_ = 3.935(1) + 0.0119(5) × *x* + 0.0119(4) × *x* ^*2*^ *; c*_*p*_ = 3.9278(8) −0.067(3) × *x* − 0.006(3) × *x* ^*2*^ *. Arrow* indicates the lattice crossover region

Structural parameters of the mixed ferrites La_1−*x*_Sm_*x*_FeO_3_ synthesized by different experimental techniques agree well with the parent LaFeO_3_ and SmFeO_3_ compounds [14, 15], as well as with the lattice parameters for Sm-doped LaFeO_3_ recently reported [24]. An analysis of the concentration dependence of unit cell dimensions in La_1−*x*_Sm_*x*_FeO_3_ series clearly proves the formation of continuous solid solution in the LaFeO_3_–SmFeO_3_ pseudo-binary system. The lattice parameters of La_1−*x*_Sm_*x*_FeO_3_ change systematically between LaFeO_3_ and SmFeO_3_ showing divergence behaviour with increasing *x*: gradual decrease of the *a-* and *c-*parameters is accompanied with detectable increasing *b* parameter (Fig. 5).

Such strongly anisotropic behaviour of the unit cell dimensions in La_1−*x*_Sm_*x*_FeO_3_ series is explained by crystal structure peculiarities of the end members of the system—LaFeO_3_ and SmFeO_3_. In spite of both compounds belong to the same GdFeO_3_-type of crystal structure (space group *Pbnm*), they show different order of the perovskite cell parameters: *b*_p_ > *a*_p_ > *c*_p_ for LaFeO_3_ and *b*_p_ > *c*_p_ > *a*_p_ for SmFeO_3_. Consequently, a crossover of *a*_p_- and *c*_p_-parameters and formation of dimensionally tetragonal structure occurs in La_1−*x*_Sm_*x*_FeO_3_ series near *x* = 0.04 (Fig. 4). Similar phenomena of the lattice parameters crossover were earlier observed in the mixed cobaltite-ferrites PrCo_1-*x*_Fe_*x*_O_3_ and NdCo_1-*x*_Fe_*x*_O_3_ [25, 26], as well as in the related rare earth aluminates and gallates *R*_1-*x*_ *R*’_*x*_AlO_3_ and *R*_1-*x*_ *R*’_*x*_GaO_3_ [27–30], in which the end members of the systems show different relations of the lattice parameters. In spite of the observed peculiarities lattice parameters behaviour, the unit cell volume in La_1−*x*_Sm_*x*_FeO_3_ series decreases almost linearly with decreasing *R*-cation radii according to the Vegard’s rule. This observation indicates statistical distribution of La and Sm species over positions of *R*-cations in La_1−*x*_Sm_*x*_FeO_3_ perovskite lattice and absence of short-range ordering in LaFeO_3_–SmFeO_3_ system.

## Conclusions

Single-phase micro- and nanocrystalline ferrites La_1−*x*_Sm_*x*_FeO_3_ with orthorhombic perovskite structure were prepared by solid state reactions (*x* = 0.2, 0.4, 0.6 and 0.8) and sol-gel citrate route (*x* = 0.5). The lattice parameters and coordinates and displacement parameters of atoms in La_1−*x*_Sm_*x*_FeO_3_ structures, as well as microstructural parameters of La_0.5_Sm_0.5_FeO_3_ nanopowders were derived from X-ray powder diffraction data by full profile Rietveld refinement technique. Obtained structural parameters of both solid state and sol-gel synthesized ferrites La_1−*x*_Sm_*x*_FeO_3_ agree well and prove the formation of continuous solid solution in LaFeO_3_–SmFeO_3_ pseudo-binary system. Peculiarity of La_1−*x*_Sm_*x*_FeO_3_ solid solution is divergence behaviour of unit cell dimensions with increasing samarium content and crossover of the *a* and *c* perovskite lattice parameters near *x* = 0.04. In comparison with a traditional energy- and time-consuming high-temperature ceramic technique, the low-temperature sol-gel citrate method is very promising tool for a synthesis of fine powders of the mixed perovskite oxide materials, free of contamination of parasitic phases.
